# Feasibility of an implementation strategy for the integration of health promotion in routine primary care: a quantitative process evaluation

**DOI:** 10.1186/s12875-017-0585-5

**Published:** 2017-02-17

**Authors:** Alvaro Sanchez, Gonzalo Grandes, Josep M. Cortada, Haizea Pombo, Catalina Martinez, Mary Helen Corrales, Enrique de la Peña, Justo Mugica, Esther Gorostiza, on behalf of the PVS group

**Affiliations:** 1 Primary Care Research Unit of Bizkaia, Basque Healthcare Service - Osakidetza, BioCruces Health Research Institute, Luis Power 18, 4ª planta, E48014 Bilbao, Spain; 2 Deusto Primary Health Care Center, Bilbao-Basurto Integrated Care Organization– Osakidetza, BioCruces Health Research Institute, Luis Power 18, E48014 Bilbao, Spain; 3 La Merced Primary Health Care Center, Bilbao-Basurto Integrated Care Organization – Osakidetza, Luis Iraurrizaga 1, 48003 Bilbao, Spain; 4 Sondika Primary Health Care Center, Uribe Integrated Care Organization – Osakidetza, Lehendakari Agirre 41, 48160 Sondika, Spain; 5 Beasain Primary Health Care Center, Goieri-Alto Urola Integrated Care Organization – Osakidetza, Bernedo s/n, 20200 Beasain, Spain; 6 Matiena Primary Health Care Center, Barrualde-Galdakao Integrated Care Organization – Osakidetza, BioCruces Health Research Institute, Trañabarren 13-Bajo, 48220 Abadiño, Spain

**Keywords:** Process evaluation, Implementation, Health promotion, Primary Health Care

## Abstract

**Background:**

Process evaluation is recommended to improve the understanding of underlying mechanisms related to clinicians, patients, context and intervention delivery that may impact on trial or program results, feasibility and transferability to practice. The aim of this study was to assess the feasibility of the Prescribe Healthy Life (PVS from the Spanish “*Prescribe Vida Saludable*”) implementation strategy for enhancing the adoption and implementation of an evidence-based health promotion intervention in primary health care.

**Methods:**

A descriptive study of 2-year implementation indicators for the PVS clinical intervention was conducted in four primary health care centers. A multifaceted collaborative modeling implementation strategy was developed to enhance the integration of a clinical intervention to promote healthy lifestyles into clinical practice. Process indicators were assessed for intervention reach, adoption, implementation, sustainability and their variability at center, practice, and patient levels.

**Results:**

Mean rates of adoption by means of active collaboration among the three main professional categories (family physicians, nurses and administrative personnel) were 75% in all centers. Just over half of the patients that attended (*n* = 11650; 51.9%) were reached in terms of having their lifestyle habits assessed, while more than a third (33.7%; *n* = 7433) and almost 10% (*n* = 2175) received advice or a printed prescription for at least one lifestyle change, respectively. Only 3.7% of the target population received a repeat prescription. These process indicators significantly (*p* < 0.001) varied by center, lifestyle habit and patient characteristics. Sustainability of intervention components changed thorough the implementation period within centers.

**Conclusions:**

The implementation strategy used showed moderate-to-good performance on process indicators related to adoption, reach, and implementation of the evidence-based healthy lifestyle promotion intervention in the context of routine primary care.

Sources of heterogeneity and instability in these indicators may improve our understanding of factors required to attain adequate program adoption and implementation through improved implementation strategies.

**Electronic supplementary material:**

The online version of this article (doi:10.1186/s12875-017-0585-5) contains supplementary material, which is available to authorized users.

## Background

In the growing field of implementation research, the process of implementation of interventions is considered important in determining how to enhance their effectiveness, feasibility and sustainability [1, 2]. Further, the development, implementation and evaluation of complex health interventions all require careful consideration not only of outcomes obtained but also of the processes involved [3]. Process evaluations within trials explore the implementation, receipt and setting of an intervention, and help interpret the outcome results and ascertain the true implications of interventions in real practice [4, 5]. Further, this type of evaluation is particularly important in multisite trials, where the “same” intervention may be implemented and received in different ways, and consequently, may make it possible to assess fidelity and to monitor tolerable intervention doses and their variability. A better understanding of program implementation may also reveal opportunities for refining intervention content and delivery [6]. Lastly, process evaluation of intervention trials may inform future implementation and roll-out of the intervention in other contexts and settings, and indicate how interventions could move from research to practice [3, 5, 6].

The Prescribe Healthy Life (PVS from the Spanish “*Prescribe Vida Saludable*”) strategy aims to optimize health promotion practice in primary and community care through implementation research [7]. Though no clinical intervention has a greater potential impact on public health than the promotion of healthy lifestyles, it remains far from being an integrated element of clinical practice, and hence, health promotion in primary care is an excellent example of the so-called “translational gap”.

PVS follows a stepwise framework, appropriate for the design, evaluation and translation of complex interventions in clinical settings [3, 8]. In 2006, a preclinical phase was carried out to characterize the problem of health promotion integration, analyzing the causes of and barriers to change as well as the theoretical basis for developing solutions [7]. Then, in 2008, we conducted the phase I or modeling phase, in which four primary health care (PHC) centers, following an implementation strategy based on a collaborative and facilitated process [9], planned and designed intervention programs adapted to their specific contexts and resources, and identified strategies for change and mechanisms through which interventions should operate. This was done collaboratively among health care professionals, researchers, health service management, community workers and public health professionals. In the present paper, we describe the phase II pilot study conducted in the same four PHC centers with the goal of piloting and optimizing the previously designed intervention programs and their assessment procedures, and exploring the adoption, implementation, sustainability, acceptance and potential effectiveness and efficiency of the intervention programs.

The aim of the process evaluation reported in this paper was to assess the feasibility of the PVS implementation strategy for integrating healthy lifestyle promotion in PHC. Within the present study, feasibility concerns to the extent, likelihood, and manner in which an evidence-based health promotion intervention has been implemented as a direct result of performing an implementation strategy aimed at enhancing the adoption of the intervention [10]. Specifically, we describe PHC population-based indicators of the reach, adoption, implementation and sustainability of a healthy lifestyle promotion intervention in the context of routine primary care, and their variability at center, practice, and patient levels. We also identify socio-demographic and clinical characteristics associated with exposure to the intervention.

## Methods

### Study design and setting

A descriptive study of 2-year implementation indicators for the PVS clinical intervention was conducted in four PHC centers. The study protocol was approved by the Primary Care Research Committee of the Basque Health Service, Osakidetza, and by the Basque Country Clinical Research Ethics Committee (Ref: 6/2009). Our health service provides universal coverage that is free at the point of use, apart from co-payment for drugs, funded through regional general taxation. Each resident is included on the list of one practice under the care of a family physician or a pediatrician and a primary care nurse, who offer comprehensive primary care and access to hospital services. Primary care professionals work in full-time PHC teams at local centers providing health care for users in a defined geographical area.

### Participants

The PVS implementation strategy has two main targets:

i) PHC centers and their staff, as the initiative seeks to improve health promotion practice through the mutual adaptation of evidence-based interventions and the center’s organization; and
ii) primary care patients attending these health centers that fail to adhere to at least one of the following lifestyle recommendations: regular physical activity, healthy diet and abstinence from smoking.

### PHC centers

Eligible centers were identified through the Medical Directors of the seven primary care organizations into which PHC is structured in the Basque Health System. Of the seven organizations, four agreed to collaborate and each selected one PHC center with the following eligibility criteria: (i) previous involvement in innovative health promotion programs or preventive practice optimization initiatives, (ii) the existence of community health promotion initiatives in their geographical area of influence, (iii) the presence of a positive attitude and cooperation among professionals, and (iv) the possibility of making changes in the organization of services. After explaining the study objectives and work plan in a meeting in each candidate center, we requested individual written commitment by a majority plus one of the staff in each of professional category (administrative personnel, nurses, family physicians, pediatricians, and others). The minimum required number of staff was obtained for all categories in all four centers. Health care professionals that committed to participate also gave written consent for the anonymous management and publication of data pertaining both to patients assigned to their practices and indicators related to their health care delivery activity.

### PHC users

As public health programs, the PVS intervention programs aim to have an impact at the whole population level. Hence, their target population was all PHC users aged 10 to 65 years old registered with any of the collaborating PHC clinicians in any of the collaborating centers that attended the center during the program implementation period (from September 2011 to September 2013). We excluded patients who moved, those diagnosed with psychotic disorders, neurological degenerative diseases, or severe mental retardation, and those in residential care or with terminal illnesses.

### Clinical intervention

The PVS clinical intervention targets three key aspects of a healthy lifestyle, namely, regular physical activity, a healthy diet and abstinence from smoking. The intervention is grounded in evidence-based theoretical models explaining behavior change: the Health Belief Model, the Theory of Planned Behaviour, the Transtheoretical model and Social-Cognitive Theory [7]. The multiple active intervention components and strategies are structured following the 5 A’s (Ask, Advise, Agree, Assist, and Arrange follow-up) intervention framework [11], as it encompasses most of the evidence-based behavior modification techniques used to promote changes in the targeted lifestyles. It is considered that this strategy is useful in primary care for its simplicity, and low requirements for training and time, as well as for the scientific evidence supporting its effectiveness [7, 11–15].

### Implementation strategy

The PVS implementation strategy worked at multiple levels: bottom-up primary care organizational change, top-down support from managers, community involvement, and the development of innovative e-health information and communication technologies [9]. Among the multiple specific implementation strategies used, three are directly related to the present study.

First, with the aim of adapting the clinical intervention to the context of each center where it was to be implemented and to redesign the health promotion delivery system, an action research-based collaborative modeling process was conducted in each of the centers with the active participation of PHC professionals and managers as well as researchers. Through a bottom-up decision making process based on discussion and consensus meetings among a multi-professional primary care team and community members guided by an external facilitator, staff in each center modeled and adapted the clinical intervention to their specific contexts and resources, and identified strategies for change, and mechanisms through which the interventions would operate. Between 10 and 13 working sessions were needed involving most of the primary care staff and at least one community-based organization. The process yielded multiple tailored healthy lifestyle intervention programs, varying between centers in the agents involved in each of the intervention actions as well as in the resources for their implementation and execution. Generally, within the collaborating PHC centers, administrative assistants, nurses and physicians were all involved in the PVS program but to different degrees. All professional categories were somewhat involved in the Assess step (A1), with considerable participation of administrative staff in order to perform the assessment before the medical consultation. Outside the center, individuals were themselves able to perform the assessment of healthy lifestyles through the PVS web questionnaire or a paper-based version of the same questionnaire delivered at community resources (e.g., company occupational health departments). The Advice and Agree steps (A2 and A3) were mainly delivered by family physicians, while the Assist step (A4) was mainly performed by nurses. All participants inside and outside the centers were involved in the Arrange follow-up (A5) process with particular involvement of administrative assistants and nurses. Based on reports by a sample of 32 professionals over 3 months of intervention piloting, the estimated mean time dedicated to each intervention component was 3, 7.84 and 13.67 min for assessing patient lifestyle (A1), and advising on (A2) and prescribing (A4) lifestyle change, respectively.

Second, an information and communication tool integrated in the electronic health record was developed in order to help and guide practitioners’ clinical decision making related to diagnosis and therapeutic interventions in promoting healthy lifestyles. The functions of this software are the following: a) to identify people whose lifestyle habits have not been assessed and recorded; b) to assess patients’ physical activity levels, diet, and smoking habits, complemented by paper-based questionnaires and a self-assessment website, c) to flag those not adhering to the recommendations; d) to identify high-priority populations for intervention based on data recorded in the electronic health records, e.g., age, motivation to change behavior, and chronic diseases; e) to guide health professionals in providing personalized and tailored advice based on effective communication of risks and benefits associated with lifestyle; f) to facilitate the prescription of plans for lifestyle modification, providing algorithms, evidence-based guidance and recommendations, warnings, timetables and information about community resources; f) to facilitate patient monitoring thorough the follow-up; and g) to integrate all the information and make it available to all staff of the center, thereby making it easier to track patients.

And lastly, there were audit and feedback strategies in the form of monthly tracking of process indicators related to patient reach and extent of implementation of intervention components. This auditing and feedback sought to maintain fidelity and intensity once initial implementation had been achieved.

### Measurements

Process indicators pertaining to program execution and user socio-demographic (age, sex, socioeconomic status) and clinical (chronic health problems) characteristics were calculated from routine data extracted from the electronic health records of Osakidetza. The Primary Care Research Unit of Bizkaia (UIAPB) in conjunction with the Informatics Department of Osakidetza were responsible for the coordination and quality control of the process and execution of the study. This research unit is expressly authorized to retrieve and use data from the electronic health records for research by the owner thereof, namely, the Governing Board of Osakidetza. The data were kept confidential, anonymized, encrypted and managed centrally, in accordance with the Spanish laws on data protection and patient autonomy.

Specifically, within the program, healthy lifestyles were assessed with the 10-item PVS-Healthy Lifestyle Screening questionnaire. Briefly, content validity of this screening questionnaire was supported by an expert panel. Further, we conducted a pilot concurrent validity study in a sample of 126 patients. Firstly, we found that the physical activity screening items were acceptably correlated with total activity counts (*r* = 0.46; *p* < 0.001) and minutes of moderate-to-vigorous activity (*r* = 0.34; *p* < 0.001) measured with an ActiGraph GT3X accelerometer. Secondly, the two items used to screen fruit and vegetable consumption were well correlated with the total (*r* = 0.50; *p* < 0.001) score on a modified version of the Mediterranean Diet Adherence Screener used in the PREDIMED study [16] and with the specific items regarding fruit and vegetable consumption (*r* = 0.54; *p* < 0.001) on the PREDIMED Food Frequency Questionnaire [17]. Thirdly, the responses obtained in the tobacco use sub-scale showed a strong correlation with the level of carbon monoxide in exhaled air (CO in ppm) measured with a breath CO monitor (*r* = 0.69, *p* < 0.001) (data not published).

Using data extracted from health records for a 4-year period, we assessed whether each of the patients had any of nine groups of chronic diseases and also checked a list of 52 conditions and specific criteria defined for each condition to consider it active, based on the work of Orueta et al. [18]. For the purposes of this study, multimorbidity was defined as the coexistence of more than one health problem in the same patient considering these 52 conditions.

A Deprivation Index (DI), defined by census tract, developed and published in 2008 [19] was used as a socioeconomic status indicator. This index is an ordinal variable, categorized into five levels (DI quintiles), providing a measure of the socioeconomic characteristics of the population of census tracts. Its design allows the estimation of socioeconomic and environmental inequalities among inhabitants by census tract in Spain. The calculation takes into account the percentages of residents in a tract who are manual workers, unemployed, temporary employees, or have an poor level of educational attainment, overall and also specifically among young people, according to the most recent census data available (2001).

To assess the effectiveness of the PVS programs in terms of public health significance we used the RE-AIM (Reach, Efficacy, Adoption, Implementation, Maintenance) framework [20]. This framework underlines that to have a public health impact programs require more than efficacy. They must reach a diverse and representative sample of the at-risk population. Their adoption in specific practice/clinical settings must be realistic. Further, programs must be able to be implemented as intended. Lastly, they must also be maintained over time in a sustainable way by the individual and the practice/clinical setting. These dimensions together determine the overall impact of a program at the population level. Specifically, the process indicators assessed within the RE-AIM dimensions are:

### Reach (Patient level)

Exposure of patients to the program at each center in terms of the percentage of the target population (patients 10 to 65 years old who attended the center at least once during the program implementation period) who had their lifestyle habits assessed, and consequently were exposed to the program.

### Adoption (Practice/Center level)

Professional participation in the program: the percentage of professionals by professional category and percentage of practices (multidisciplinary health care professionals with an assigned list of patients, e.g., family physician plus nurse) in each center participating in the program who had performed at least one intervention component.

### Implementation (Practice/Center level)

The percentage of practices that assessed at least 50% of their target population, gave advice to 25% and prescribed lifestyle change with a personalized plan to 10%.

### Implementation (Patient level)

Reception of intervention components as intended in terms of the percentage of target patients whose lifestyle habits were assessed and included because they failed to adhere to at least one healthy lifestyle recommendation (I1: Included); the percentages of target patients (I2a: Advised-target) and included patients (I2b: Advised-included) who received advice; the percentages of target patients (I3a: Assisted-target), included patients (I3b: Assisted-included), and advised patients (I3c: Assisted-advised) who received a prescription for lifestyle change; and the percentages of target patients (I4a: Arranged-target), included patients (I4b: Arranged-included), and prescribed patients (I4c: Arranged-prescribed) who received a repeat prescription related to the same aspect of their lifestyle.

### Maintenance

Sustainability of intervention program components over time in terms of the monthly rates of the aforementioned process indicators over 2 years.

Lastly, we also explored the association of patient socio-demographic and clinical characteristics with the likelihood of being assessed, advised or prescribed lifestyle changes.

### Analysis

Data analysis was performed with SAS software. Frequencies and proportions were used to describe professional, practice and patient characteristics for continuous and categorical variables, respectively. Process indicators for each collaborating center and in total regarding adoption, reach and implementation were expressed as frequencies and percentages along with their corresponding 95% confidence intervals (CIs). Survival analysis was used to describe sustainability and compare the incidence of exposure to the intervention components (time until exposure to lifestyle assessment, advice, and prescription for lifestyle change) between the different centers. For univariate analysis of the association of time until exposure to the different intervention components with the comparison centers, cumulative survival probabilities over 24 months were estimated and compared using Kaplan-Meier curves along with the log-rank and Wilcoxon tests (SAS PROC LIFETEST, version 9.2, SAS Institute; 2008). Time to event or censoring was defined as the time between study entry (date of baseline interview) and date of exposure to the specific intervention component, or possible censoring at the end of the study (September 19, 2013), respectively.

Adjusted hazard ratios (AHRs) and 95% CIs describing relations between patient characteristics and the exposure to the intervention components were estimated using Cox proportional hazards models [21]. These models included health care center, sex, age, recorded chronic diseases, and the deprivation index as independent factors. The proportional hazard assumption was tested, introducing the interaction of each of the variables with time in the models. In the case of covariates for which the proportional hazard assumption was not satisfied, the Cox model was extended with time-dependent variables allowing hazard ratios to change over time.

## Results

### Practitioner team level (Center/professional level)

#### Adoption, Implementation & Maintenance

Table 1 describes the size and composition of the four collaborating PHC centers. Briefly, center size ranged from 14 to 36 staff mainly consisting of family physicians (range: 4 to 13), practice nurses (range: 5 to 13), pediatricians (range: 1 to 2) and administrative personnel (range: 4 to 6). Other professional categories included midwives, dentists and family planning physicians. Overall, 68 out of a total of 90 staff (75%) across professional categories (administrative personnel, nurses, family physicians, pediatricians, and others) initially committed in writing to participate in the study. This initial collaboration rate varied between centers ranging from 68 to 100% (*p* < 0.01). Within the centers, family physicians followed by nurses and administrative personnel were the most strongly represented categories, while pediatricians and especially midwives had the lowest rates of collaboration. As final unit of observation at center/professional level, we considered practices: the initial collaboration within centers ranged from 63 to 100% of practices with an overall participation of 84% from a total of 32.

**Table 1:** Professional and practice level participation, and patient demographic and clinical characteristics, by center

	Center 1	Center 2	Center 3	Center 4	Total
Professional level					
Family physicians	11/13	5/6	3/4	4/4	23/27 (85%)
Nurses	9/13	6/8	4/5	6/6	25/32 (78%)
Administrative personnel	5/6	2/5	3/4	4/4	14/19 (74%)
Pediatricians	0/2	2/2	0/1	1/1	3/6 (50%)
Midwifes	0/2	0/1	−	1/1	1/4 (25%)
Others^a^	−	−	−	2/2	
Total	25/36 (69%)	15/22 (68%)	10/14 (71%)	18/18 (100%)	68/90 (75%)
Practice level					
Initial commitment	11/14	7/8	4/4	5/5	27/32 (84%)
Assessed (A1) 50% of target patients	2/11	4/7	2/4	5/5	13/27 (48%)
Advised (A2) 25% of target patients	4/11	5/7	1/4	5/5	15/27 (55%)
Assisted (A4) 10% of target patients	3/11	3/7	2/4	5/5	13/27 (48%)
Patient level					
Male sex	5126 (49.4%)	3215 (54.6%)	1266 (49.3%)	1760 (48.4%)	11367 (50.6%)
	(48.4%–50.4%)	(53.4%–55.9)	(47.4%–1.2%)	(46.8%–50.0%)	(49.9%–51.3%)
Age, years					
10–19	273 (2.6%)	551 (9.4%)	373 (14.5%)	367 (10.1%)	1564 (7%)
	(2.3%–2.9%)	(8.6%–10.1%)	(13.2%–5.9%)	(9.1%–11.15)	(6.6%–7.3%)
20–45	5713 (55.1%)	3585 (60.9%)	1214 (47.3%)	1881 (51.7%)	12393 (55.2%)
	(54.1%–56.0%)	(59.7%–62.2%)	(45.3%–9.2%)	(50.1%–3.4%)	(54.5%–55.8%)
46–65	4387 (42.3%)	1747 (29.7%)	981 (38.2%)	1387 (38.2%)	8502 (37.9%)
	(41.3%–43.2%)	(28.5%–30.8%)	(36.3%–40.1%)	(36.6%–9.7%)	(37.2%–38.5%)
Health problems					
Cancer^b^	215 (2.2%)	88 (1.6%)	41 (1.6%)	65 (1.8%)	409 (1.9%)
	(1.9%–2.4%)	(1.3%–2.0%)	(1.4%–2.3%)	(1.1%–2.2%)	(1.7%–2.1%)
Neurological disorder^a^	377 (3.8%)	206 (3.8%)	80 (3.2%)	165 (4.6%)	828 (3.9%)
	(3.4%–4.2%)	(3.4%/4.4%(	(2.5%–3.9%)	(3.9%–5.3%)	(3.6%–4.1%)
Cardiovascular disease^a^	1371 (13.8%)	610 (11.4%)	268 (10.8%)	481 (13.5%)	2730 (12.8%)
	(13.1%–14.5%)	(10.6%–12.3%)	(9.6%–12.1%)	(12.4%–14.6%)	(12.3%–13.2%)
Musculoskeletal disorder^a^	637 (6.4%)	311 (5.8%)	121 (4.9%)	268 (7.5%)	1337 (6.3%)
	(5.9%–6.9%)	(5.2%–6.4%)	(4.0%–5.7%)	(6.7%–8.4%)	(5.9%–6.6%)
Mental health problem^a^	1415 (14.2%)	1032 (19.3%)	357 (14.4%)	647 (18.2%)	3451 (16.2%)
	(13.5%–14.9%)	(18.2–20.4%)	(13.0%–15.8%)	(16.9%–19.5%)	(15.7%–16.7%)
Respiratory disease^a^	398 (4.0%)	342 (6.4%)	116 (4.7%)	211 (5.9%)	1067 (5.0%)
	(3.6%–4.4%)	(5.7%–7.0%)	(3.8%–5.5%)	(5.2%–6.7%)	(4.7%–5.3%)
Digestive disease^a^	415 (4.2%)	259 (4.8%)	84 (3.4%)	153 (4.3%)	911 (4.3%)
	(3.8%–4.6%)	(4.3%–5.4%)	(2.7%–4.1%)	(3.6%–5.0%)	(4.0%–4.5%)
Metabolism-related disease^a^	873 (8.7%)	383 (7.2%)	143 (5.8%)	257 (7.2%)	1656 (7.8%)
	(8.2%–9.3%)	(6.5%–7.8%)	(4.8%–6.7%)	(6.4%–8.1%)	(7.4%–8.1%)
Other^a^	621 (6.2%)	449 (8.4%)	157 (6.3%)	250 (7.0%)	1477 (6.9%)
	(5.8%–6.7%)	(7.6%–9.1%)	(5.4%–7.3%)	(6.2%–7.9%)	(6.6%–7.3%)
No chronic health problems	5885 (59.2%)	3098 (57.9%)	1560 (62.9%)	1954 (54.9%)	12497 (58.6%)
	(58.2%–60.2%)	(56.6%–59.3%)	(61.0%–64.8%)	(53.3%–56.6%)	(57.9%–59.3%)
Deprivation Index					
I	48 (0.5%)	230 (4.3%)	536 (21.4%)	87 (2.4%)	901 (4.2%)
	(0.3%–0.6%)	(3.7%–4.8%)	(19.8%–3.0%)	(1.9%–2.9%)	(3.9%–4.5%)
II	3456 (34.8%)	176 (3.3%)	1848 (73.8%)	2094 (58.8%)	7574 (35.4%)
	(33.8%–35.7%)	(2.8%–3.8%)	(72.1%–5.5%)	(57.2%–60.4%)	(34.8%–36.1%)
III	4867 (48.9%)	151 (2.8%)	43 (1.7%)	46 (1.3%)	5107 (23.9%)
	(48.0%–49.9%)	(2.4%–3.2%)	(1.2%–2.2%)	(0.9%–1.7%)	(23.3%–24.5%)
IV	907 (9.1%)	167 (3.1%)	35 (1.4%)	289 (8.1%)	1398 (6.6%)
	(8.5%–9.7%)	(2.6%–3.6%)	(0.9%–1.8%)	(7.2%–9.0%	(6.2%–6.95)
V	664 (6.7%)	4637 (86.5%)	43 (1.7%)	1043 (29.3%)	6387 (29.9%)
	(6.2%–7.2%)	(85.6%–87.4%)	(1.2%–2.2%)	(27.8%–30.8%)	(29.3%–30.5%)
Failure to adhere to ≥1 lifestyle recommendation	3206 (84.5%)	3070 (95.7%)	1536 (93.7%)	2693 (89.5%)	10505 (90.2%)
Insufficient physical activity	1979 (53.1%)	2250 (70.8%)	728 (61.9%)	1570 (58.3%)	6527 (60.6%)
Insufficient fruit and vegetable consumption	2507 (67.1%)	2766 (86.9%)	888 (76.6%)	1888 (70.1%)	8049 (74.7%)
Smoker	1009 (27.3%)	1146 (36.6%)	541 (33.8%)	978 (32.6%)	3674 (32.1%)

^a^Dentist and family planning physician

^b^ *N* = 21323

Implementation of program components also varied between practices and centers. Specifically, rates of participation of practices in the execution of program components were not similar, except in one of the centers in which all teams collaborated and worked equally well. Overall, only 48% of practices assessed the lifestyles of 50% of all target patients that attended the center at least once during the program implementation period. Within centers, this rate of assessment was achieved by half or more than half of practices in three of the centers. However, only in two of the centers did half or more than half of collaborating practices reach levels of 25% or 10% of target patients being advised or prescribed lifestyle changes, respectively.

### Patient level

#### Reach, Implementation & Maintenance

A total sample of 22459 target patients aged 10 to 65 years old assigned to collaborating practices attended their PHC center at least once during program implementation period. As presented in Table 1, overall, half of the target patients were male (50.6%) and more than a half (55.2%) were young adults 20 to 45 years of age, with 37.9% and 7% of target patients being adults and adolescents, respectively. Approximately 40% of target patients had at least one chronic condition. The most prevalent diagnosed chronic health problems were mental health problems (16.2%) followed by cardiovascular (12.8%) and metabolism- related (7.8%) diseases.

Within centers, a higher percentage of women were present in the sample in center 2, and the age distribution of primary care users was slightly older in center 1. The most distinct characteristic of the populations was socioeconomic status (Table 1), with one of the centers having the vast majority of users in the lowest status (86.5%), while in two other centers users were mostly included in the three highest categories, and in the fourth center users were mostly in the second (59%) and lowest (29%) socioeconomic status categories.

Of the total sample of patients who attended the centers, 11650 (51.9%) had their lifestyle habits assessed (Table 2). The percentage of target patients reached ranged from 36.6 to 82.8% in collaborating centers. Among patients whose lifestyles were assessed, 10505 (90.2% of those assessed) did not adhere to at least one of the three healthy lifestyle recommendations considered and were therefore included in the intervention program (I1; see Table 1). Specifically, 32.1% of patients assessed and found to be eligible for the intervention were smokers, 74.7% did not consume five servings of fruits and vegetables a day, and 60.6% did not meet the recommended levels of aerobic physical activity (moderate intensity physical activity for ≥30 min 5 day/week or vigorous intensity activity for ≥20 min 3 day/week, or a combination of the two). The other 1145 (9.8%) adhered to recommendations on all three healthy lifestyles and were excluded from receiving the intervention. Among the collaborating centers, center 2 had the highest cumulative prevalence of unhealthy lifestyles.

**Table 2:** Implementation indicators at target and included patient population levels, by center and lifestyle habit

	Center 1	Center 2	Center 3	Center 4	Total
*Target population*	10373	5883	2568	3635	22459
A1 at least on 1 lifestyle	3792 (36.6%)	3209 (54.5%)	1640 (63.7%)	3009 (82.8%)	11650 (51.9%)
	(35.6–37.5%)	(53.3–55.8%)	(62.0–65.7%)	(81.5–84.0%)	(51.2–52.5%)
A2 at least on 1 lifestyle	2318 (22.3%)	2170 (36.9%)	809 (31.5%)	2136 (58.7%)	7433 (33.1%)
	(21.5–23.1%)	(35.6–38.1%)	(29.7–33.3%)	(57.2–60.4%)	(32.5–33.7%)
A4 at least on 1 lifestyle	840 (8.1%)	406 (6.9%)	377 (14.7%)	552 (15.2%)	2175 (9.7%)
	(7.6–8.6%)	(6.2–7.5%)	(13.3–16.1%)	(14.0–16.3%)	(9.3–10.1%)
A5 at least on 1 lifestyle	231 (2.2%)	198 (3.4%)	177 (6.9%)	218 (6.0%)	824 (3.7%)
	(1.9–2.5%)	(2.95–3.8%)	(5.9–7.9%)	(5.2–6.7%)	(3.4–3.9%)
Physical Activity (PA)					
A1_PA	3727 (35.9%)	3176 (54%)	1176 (45.8%)	2692 (74.1%)	10771 (48.0%)
	(35.0–36.8%)	(52.7–55.3%)	(43.9–47.7%)	(72.6–75.5%)	(47.3–48.6%)
A2_PA	1097 (10.6%)	1148 (19.5%)	386 (15%)	1071 (29.5%)	3702 (16.5%)
	(10.0–11.2%)	(18.5–20.5%)	(13.6–16.4%)	(28.0–30.9%)	(16.0–17.0%)
A4_PA	315 (3%)	123 (2.1%)	161 (6.3%)	194 (5.3%)	793 (3.5%)
	(2.7–3.4%)	(1.7–2.4%)	(5.3–7.2%)	(4.6–6.1%)	(3.3–3.8%)
A5_PA	50 (0.5%)	38 (0.6%)	68 (2.6%)	43 (1.2%)	199 (0.9%)
	(0.3–0.6%)	(0.4–0.8%)	(2.0–3.2%)	(0.8–1.5%)	(0.8–1.0%)
Diet (DT)					
A1_DT	3737 (36%)	3182 (54.1%)	1159 (45.1%)	2693 (74.1%)	10771 (48.0%)
	(35.1–36.9%)	(52.8–55.4%)	(43.2–47.0%)	(72.7–75.5%)	(47.3–48.6%)
A2_DT	1688 (16.3%)	1848 (31.4%)	486 (18.9%)	1407 (38.7%)	5429 (24.2%)
	(15.6–17.0%)	(30.2–32.6%)	(17.4–20.4%)	(37.1–40.3%)	(23.6–24.7%)
A4_DT	549 (5.3%)	319 (5.4%)	217 (8.5%)	353 (9.7%)	1438 (6.4%)
	(4.9–5.7%)	(4.8–6.0%)	(7.4–9.5%)	(8.7–10.7%)	(6.1–6.7%)
A5_DT	154 (1.5%)	176 (3%)	110 (4.3%)	144 (3.9%)	584 (2.6%)
	(1.2–1.7%)	(2.5–3.4%)	(3.5–5.1%)	(3.3–4.6%)	(2.4–2.8%)
Tobacco (TB)					
A1_TB	3698 (35.6%)	3133 (53.3%)	1599 (62.3%)	2997 (82.4%)	11427 (50.9%)
	(34.7–36.6%)	(52.0–54.5%)	(60.4–64.1%)	(81.2–83.7%)	(50.2–51.5%)
A2_TB	652 (6.3%)	836 (14.2%)	227 (8.8%)	676 (18.6%)	2391 (10.6%)
	(5.8–6.7%)	(13.3–15.1%)	(7.7–9.9%)	(17.3–19.7%)	(10.2–11.0%)
A4_TB	99 (1%)	60 (1%)	40 (1.6%)	67 (1.7%)	266 (1.2%)
	(0.8–1.1%)	(0.8–1.3%)	(1.1–2.0%)	(1.4–2.3%)	(1.0–1.3%)
A5_TB	42 (0.4%)	17 (0.3%)	4 (0.2%)	34 (0.9%)	97 (0.4%)
	(0.3–0.5%)	(0.1–0.4%)	(0.0–0.3%)	(0.6–1.2%)	(0.3–0.5%)

Note: A1 refers to Assess; A2 refers to Advise; A4 refers to Assist with a prescription for change; and A5 refers to arrange follow-up with a repeat prescription

Advice on change in at least one of the lifestyle habits was provided to a third (33.7%; *n* = 7433) of all target patients (I2a) and to 70.7% of included patients found not to adhere to at least one lifestyle recommendation when assessed (I2b) (Table 2). Regarding the areas in which advice was given, diet was the lifestyle on which the highest percentage of patients received advice (24.2% of target patients; 67.4% of those not eating five servings of fruits and vegetables a day). Advice for increasing physical activity was provided to 16.5% of target patients and 56.7% of those not reaching recommended physical activity levels, while advice on smoking cessation was given the least in terms of reaching target patients (10.6% of target patients; 65.1% of those identified as smokers at inclusion).

Regarding lifestyle prescription, 9.7% (*n* = 2175) of all target patients collaboratively designed and then received a printed prescription for at least one healthy lifestyle change (I3a), diet being the lifestyle most frequently addressed (6.4% of all target patients) followed by physical activity and smoking cessation (3.5% and 1.25% of all target patients, respectively). From those included in the program with unhealthy lifestyles, 20.7% received a prescription for at least one lifestyle change (I3b), while 29.3% of those who received advice regarding lifestyle changes finally ended up designing and receiving a personalized lifestyle change plan (I3c). Further, of those who received dietary advice, 26.5% were also prescribed dietary changes, while prescriptions regarding increasing physical activity and smoking cessation were provided to 21.4% and 11.1% of those advised, respectively.

Only 3.7% of the target population received repeat prescriptions and consequently the full 5 A’s intervention over the course of the program (I4a), again with diet being the lifestyle addressed in most cases (2.6% for diet; 1% for physical activity and 0.4% for smoking cessation). The percentage of patients with a repeat prescriptions rose to 7.8% and 37.9% in included patients’ (I4b) and in those with a previous prescription (I4c), respectively. Multiple-lifestyle intervention indicators were as follows: 4.7% of the target population (*n* = 1057) received advice on all three lifestyles considered and 12.7% (*n* = 2861) received it on two. This corresponded to only 35 patients who attended the centers (0.15%) receiving a prescription to change physical activity, diet and smoking habits, while in 609 (2.7%) modifications in two aspects of patient lifestyles were planned and prescribed.

**Fig. 1:**
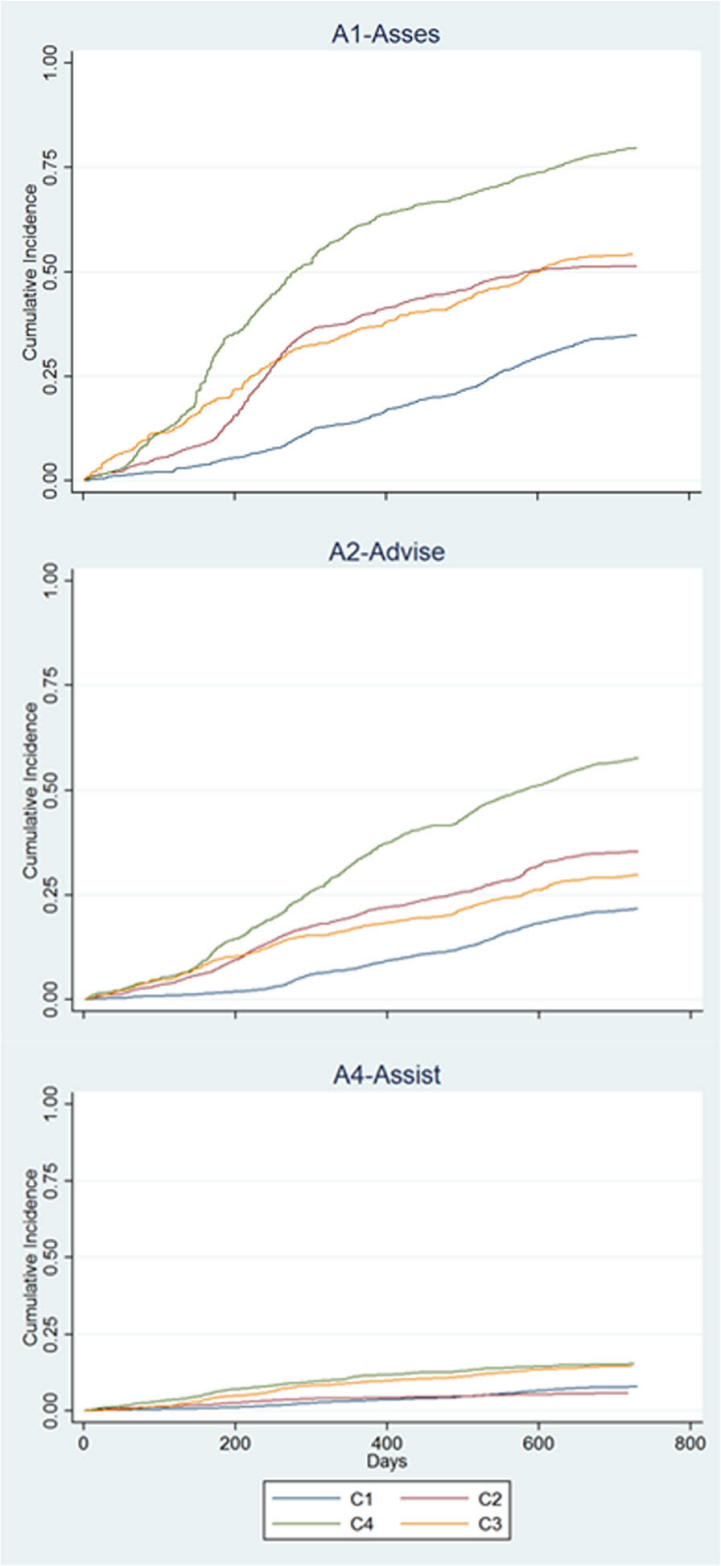
Cumulative exposure of patients to the main intervention components in the four collaborating centers over the 2-year program implementation period

Figure 1 illustrates the cumulative exposure of patients to the main intervention components in the four collaborating centers over the 2-year program implementation period. There was a significant between-center difference in the monthly rate for assessment (A1), advice (A2) and prescription of a healthy lifestyle change (A4) (Log-rank test of equality for survival distributions < 0.001). Furthermore, ‘center’ did not satisfy the proportionality assumption (*P* < 0.001 for the interaction between center and time) and hence was included in an extended Cox model as a time-dependent variable (Table 3). As an example, during the first 3 months, compared to the reference center (center 1), exposure to assessment was 3.42, 8 and 6 times more likely in centers 2, 3 and 4, respectively. Patients of these three centers were more than 4 times more likely than those in center 1 to be exposed to advice, and 2 to 4.22 times more likely to receive a prescription for lifestyle change.

**Table 3:** Characteristics of target population associated with exposure to the main intervention components: Assessment of lifestyles (A1), Advice on lifestyle change (A2), and Prescription of lifestyle change (A4)

Variables			Adjusted Hazard Ratio (95% CI)		
			Assessed (A1)	Advised (A2)	Prescribed (A4)
Age (unit = 5 years)			1.05 (1.04–1.06)	1.04 (1.03–1.05)	1.04 (1.02–1.06)
Sex		Female	1.00	1.00	1.00
		Male	0.83 (0.80–0.86)	0.84 (0.80–0.88)	0.68 (0.63–0.74)
Cardiovascular disease		No	1.00	1.00	1.00
		Yes	1.41 (1.33–1.49)	1.51 (1.42–1.62)	1.87 (1.67–2.09)
Musculoskeletal disorder		No	1.00	1.00	1.00
		Yes	1.19 (1.11–1.28)	1.15 (1.05–1.25)	1.20 (1.04–1.39)
Mental health problem		No	1.00	1.00	1.00
		Yes	1.09 (1.04–1.14)	1.18 (1.11–1.25)	1.15 (1.03–1.28)
Respiratory disease		No	1.00	1.00	1.00
		Yes	1.25 (1.15–1.35)	1.36 (1.24–1.49)	1.51 (1.29–1.77)
Digestive disease		No	1.00	1.00	1.00
		Yes	1.12 (1.03–1.23)	1.12 (1.01–1.24)	1.25 (1.06–1.49)
Metabolism-related disease		No	1.00	1.00	1.00
		Yes	1.25 (1.17–1.34)	1.26 (1.16–1.36)	1.32 (1.16–1.51)
Other chronic health problem		No	1.00	1.00	--
		Yes	1.13 (1.05–1.21)	1.14 (1.05–1.24)	--
Deprivation Index		High	1.00	1.00	1.00
		Medium	1.18 (1.10–1.27)	1.17 (1.10–1.25)	1.14 (0.98–1.33)
		Low	1.16 (1.10–1.22)	1.19 (1.11–1.27)	1.25 (1.11–1.42)
Center		Center 1	1.00	1.00	1.00
	0–3 months	Center 2	3.42 (3.00–3.90)	4.23 (3.47–5.16)	3.24 (2.47–4.25)
		Center 3	8.54 (7.54–9.66)	5.04 (4.07–6.25)	2.06 (1.46–2.89)
		Center 4	6.19 (5.48–7.00)	4.54 (3.70–5.56)	4.22 (3.25–5.47)
	3–6 months	Center 2	2.97 (2.52–3.48)	7.43 (5.77–9.56)	2.62 (1.78–3.88)
		Center 3	3.55 (2.93–4.30)	6.91 (5.22–9.15)	6.55 (4.57–9.38)
		Center 4	8.96 (7.78–10.32)	10.59 (8.27–13.57)	6.38 (4.54–8.97)
	6–12 months	Center 2	4.42 (4.02–4.85)	2.81 (2.49–3.18)	0.95 (0.73–1.23)
		Center 3	2.37 (2.08–2.70)	1.43 (1.20–1.69)	2.16 (1.71–2.73)
		Center 4	5.69 (5.17–6.27)	4.45 (3.97–4.99)	2.03 (1.64–2.52)
	12–24 months	Center 2	1.01 (0.92–1.12)	1.52 (1.37–1.68)	0.41 (0.32–0.53)
		Center 3	1.26 (1.12–1.42)	1.01 (0.89–1.15)	1.24 (1.01–1.52)
		Center 4	2.50 (2.27–2.75)	2.91 (2.65–3.19)	0.91 (0.75–1.11)

With respect to the other variables included in the model, most patient personal and clinical characteristics where also associated with the implementation of the different intervention components (Table 3). Specifically, the hazard ratio for exposure to components of the healthy lifestyle promotion intervention was higher in women than in men (AHR of being assessed in men: 0.83; 95% CI: 0.80–0.86) and increased with patient age, 1.05-fold for every 5 years of age. Some of the chronic conditions were also related to exposure to intervention components. The hazard ratio for exposure to assessment, advice and prescription for lifestyle change was significantly higher in those with cardiovascular, musculoskeletal, respiratory, digestive, and metabolism-related diseases, among other conditions, than in patients without these conditions. Among these chronic health problems, cardiovascular diseases had the strongest association with being assessed (AHR: 1.41; 95% CI: 1.33 to 1.49), advised (HR: 1.51; 95% CI: 1.42 to 1.62), and receiving a prescription for lifestyle change (AHR: 1.87; 95% CI: 1.67 to 2.09).

## Discussion

Results of this pilot study showed that the PVS implementation strategy was feasible and achieved modest-to-good improvement in clinical practice regarding adoption, reach, and implementation of an evidence-based healthy lifestyle promotion intervention in the context of routine primary care. The main contribution of this study is the description of the significant heterogeneity observed in the assessed process indicators associated with lifestyle habits, patient characteristics, settings and implementation timeframe.

Overall, rates of adoption within centers were high in the three main professional categories considered, family physicians, nurses and administrative personnel. During the 2-year period of program implementation, more than the half of all the primary care users aged 10 to 65 years old were reached in the four centers in terms of having their lifestyles assessed. This level of reach should be considered notably high, given that the program has been implemented in “real world” conditions in primary care, among those attending routine appointments, and thus represents general and unselected sample of the primary care population for the groups covered. Other studies evaluating the feasibility of lifestyle screening in routine context have attained reach rates ranging from 49.6 to 86.7%, though these figures have been attained in shorter exposure periods or/and with limited sample sizes [22–24].

Research on healthy lifestyle promotion in clinical settings has established that intensive counseling in the form of advice and the use of behavioral change techniques are some of the active components that help individuals initiate and maintain health-related behavior change [7]. The PVS implementation strategy aimed to enhance the integration and feasibility of the execution of these effective intervention components [9]. PVS centers have been able to provide advice on change in at least one of the lifestyles considered to a third of patients who attended the centers and almost 10% of these patients have collaboratively designed and then received a printed prescription for at least one lifestyle change. Other implementation trials aiming to improve the promotion of healthy lifestyles have also shown positive results in terms of increasing screening and intervention rates [25–29]. However, as observed in other studies [30, 31], a full 5 A’s intervention is rarely performed, only 4% of patients receiving all components in relation to at least one aspect of their lifestyle.

These process and performance indicators regarding adoption, reach and implementation varied between lifestyle habits, patient characteristics and centers. First, regarding lifestyle habits, higher percentages of patients received advice and a prescription for change in the case of physical activity and diet once these had been assessed, while smoking habits has been the lifestyle most commonly assessed but with the lowest rates of advice and prescription. This variability in the priority given to the healthy lifestyles was a result of the implementation strategy used, which sought to help collaborating centers adapt the intervention program to their actual context and resources in order to enhance its integration in routine service delivery in PHC. In this adaptation process, they were instructed to prioritize both the lifestyles to be addressed and strategies to re-organize the delivery of preventive actions within centers. Considering that 90% of patients assessed in this study failed to adhere to at least one of the lifestyle recommendations considered, it seems reasonable to apply some sort of prioritization strategy to deal with work overload and maximize feasibility of a population-level healthy lifestyle promotion program in the context of routine primary care. Further research is warranted as to identify what are the best cut-off points for attaining the optimal balance between feasibility and workload when identifying individuals in need of a healthy lifestyle promotion intervention.

Second, the delivery of health promotion actions has been found to be associated with various personal and clinical characteristics of the targeted population. Regarding patients’ intra-personal characteristics, the likelihood of being assessed, receiving advice and being prescribed lifestyle changes increased with age and was higher in women than men. Those with chronic health conditions (e.g., cardiovascular disease) were also more likely to receive each of the intervention components. A possible explanation for these associations is that health care providers have targeted and prioritized their healthy lifestyle promotion actions to those more likely to face fewer obstacles to changing their lifestyle, such as, older patients who may have more free time (as they are working less or retired completely and have fewer obligations); or to those that would potentially benefit the most, such as those with a chronic health problem. Indeed, selective and targeted counseling of patients prioritizing among other factors patient with known health risks or readiness for change has been documented [32] and is currently recommended in order to maximize their impact [33, 34].

Moreover, factors beyond personal characteristics, such as socioeconomic status also influenced the implementation of intervention components. Specifically, each of the main intervention components were more likely to be received by those with higher socioeconomic status. Therefore, it seems that intra- and extra-personal characteristics of target populations may also explain variability in the integration and implementation of healthy lifestyle promotion and the feasibility of this type of intervention [32, 35].

Third, PHC center has been observed to be a great source of variability in program adoption, reach and implementation indicators. Overall, relatively high rates of adoption by staff have been attained in all the centers. This is not surprising given that written commitment from the majority of staff within the different categories was a requirement for study participation. However, actual rates of involvement and contribution differed: while in one of the centers all staff committed to participate and did so, in other centers complete participation was not attained. These rates of adoption may have a direct impact on both the actual reach of the program and the implementation of intervention components as could be observed in the present study. On the one hand, in the center in which all staff participated, more than 80% of the target population was reached in terms of the identification of at least one healthy lifestyle, and almost 60% received advice on lifestyle changes, as well as the highest percentage of target users (comparing with other centers) received a prescription of a lifestyle change plan. Additionally, within collaborating professionals, all of them actually showed good performance in terms of indicators of implementation. On the other hand, the center with the lowest rates of adoption obtained the poorest rates of implementation of intervention components at practices and patient levels (e.g., 37% of target patients assessed and only 2 out of 11 collaborating practices assessed at least 50% of target users seen during the program implementation period).

Furthermore, rates of adoption by staff and reach at the patient level may have contributed substantially to the observed variability in implementation rates at the center level. Specifically, the likelihood of being exposed to intervention components of advice and prescription for lifestyle change was 4 times more likely in the center with the highest rates of adoption and reach than the center with the lowest rates of adoption and reach. The likelihood of exposure to the intervention program in the other two centers with intermediate rates of adoption and reach was also significantly higher than in the center with the lowest rates. In this sense, based on the actual adoption and implementation of intervention components across collaborating health centers, we may distinguish three levels of implementation performance: high, medium and low.

Variability in program adoption by professionals, program reach and implementation indicators between different sites has also been reported in other implementation trials [24, 36]. Centers each have different contexts and thus implementation strategies must be targeted and adapted to those specific contexts. Even after a hypothetical adaptation, results and products would be context specific. That is, context is crucial, meaning that interventions may be implemented in different ways in different settings and thus outcomes may also vary [37–39].

And finally, another important finding of the present study is that the sustainability or the extent of exposure to intervention components over time differed between centers and varied thorough the implementation period within centers, probably reflecting intermittent efforts and continuous adaptation to the multiple unstable contextual and organizational factors in the centers, to the innovation itself, the providers, the processes involved and the interaction of all of these factors [40–42]. We should recognize that in the present implementation study competition with other initiatives, in particular those imposed by health system management, structural changes and staff movements, lack of ongoing training of staff, and technical problems with the information and communication tool may all have hindered ideal implementation of intervention components throughout the implementation period [see accompanying paper by Martinez et al.].

The present study has several limitations. First, the process evaluation refers to a prospective cohort study performed in four selected centers and the observational nature of the study together with the absence of a comparison group mean that it is not possible to attribute the results obtained to the implementation strategy alone. Although collaborating centers are apparently diverse, they may not be representative of all PHC centers in our health service. In addition, though we have described some characteristics regarding representativeness (in terms of PHC size and composition, socioeconomic status of catchment populations, and so on), missed information regarding other specific characteristics of the context may hinder interpretation of results. Though the external validity of the study may be questioned, implementation trials aim to conduct a broad evaluation of the translation of proven-efficacy interventions into routine care, assessing results in heterogeneous, unselected populations and real-life clinical settings [43, 44]. Another limitation relates to difficulties interpreting the results obtained, especially those concerning variability between centers/settings and the nature of changes or reasons for intermittent sustainability. Consequently, a qualitative inquiry study has also been performed to explore staff perceptions of the program implementation and its results [see accompanying paper by Martinez et al.].

The main strengths of the study are that it has been conducted at the population level and the quality of data assessed, as these were recorded in and subsequently retrieved from an established electronic health record.

## Conclusions

Implementation research must be the priority in order to first transform our health systems into continuously learning organizations and consequently enact the final step of translating scientific knowledge to real-life clinical settings in an effort to close the translational gap. In doing so, engagement of all stakeholders is crucial, especially of those who interact with patients and are the actual providers of health care. However, contexts are heterogeneous and unstable and an adequate program adoption and implementation may not always be achieved. Identifying the factors behind implementation heterogeneity may help us to tailor implementation strategies, but comparative effectiveness research is needed to determine the best theoretically grounded and operationalized implementation strategies to improve clinical practice.

## Additional file

Additional file 1: Appendix 1. Members of the PVS group. (PDF 192 kb)

## Abbreviations

5 A’s: Ask, Advise, Agree, Assist, and Arrange follow-up
A1: Assessment of healthy lifestyles
A2: Advice on healthy lifestyle change
A4: Prescription of a healthy lifestyle change
AHR: Adjusted hazard ratio
CI: 95% confidence interval
DI: Deprivation Index
I1: Included: percentage of target patients whose lifestyle habits were assessed and included because they failed to adhere to at least one healthy lifestyle recommendation
I2a: The percentage of target patients who received advice
I2b: The percentage of included patients who received advice
I3a: The percentage of target patients who received a prescription for lifestyle change
I3b: The percentage of included patients who received a prescription for lifestyle change
I3c: The percentage of advised patients who received a prescription for lifestyle change
I4a: The percentage of target patients who received a repeat prescription related to the same aspect of their lifestyle
I4b: The percentage of included patients who received a repeat prescription related to the same aspect of their lifestyle.
I4c: The percentage of prescribed patients who received a repeat prescription related to the same aspect of their lifestyle.
PHC: Primary Health Care
PREDIMED: *Prevención con Dieta Mediterranea* study
PVS: *Prescribe Vida Saludable* study
RE-AIM: Reach, Efficacy, Adoption, Implementation, Maintenance framework
UIAPB: The Primary Care Research Unit of Bizkaia
