# Supplementary material for: Feasibility of an implementation strategy for the integration of health promotion in routine primary care: a quantitative process evaluation
Source: BMC Fam Pract. 2017 Feb 17;18:24. doi: 10.1186/s12875-017-0585-5 (PMC5316192; doi:10.1186/s12875-017-0585-5)
Supplement: Additional file 1: Appendix 1. — Members of the PVS group. (PDF 192 kb) [file 12875_2017_585_MOESM1_ESM.pdf]

## Appendix 1: Members of the PVS Group

Research Team: *Primary Care Research Unit of Bizkaia, Basque Health Service–Osakidetza*: principal investigator: Gonzalo Grandes; co-investigators: Alvaro Sanchez, Haizea Pombo, Josep M Cortada, Catalina Martinez, Paola Bully, Aitor Sanz-Guinea).

Advisory Committee

Maribel Cifuentes (university of Colorado, School of Medicine, Department od Family Medicine); Gonzalo Bacigalupe (University of Massachussetts Boston, College os Education ans Human Developement); Marie-Pierre Gagnon (Université Laval, Faculty of Nursing Sciences).

Basque Health Service–Osakidetza:

*Deputy Director of Healthcare Services*: Carlos Sola

*Department of Information Technology*: Martin Begoña, Susana Iglesias, Maite Cuadrado, Nuria Gonzalez.

*Management of Goierri-Alto Urola District*: Teresa Garmendia; M Luz Jauregui; Amaia Hernando. *Beasain Health Center*: Justo Mugica; M Pilar Alberdi, M Angeles Arrondo, Amaia Azkoitia, Xabier Epaizabal, M Aranzazu Echeverria, M Esperanza Garcia, M Angeles Garcia, M Erkuden Imaz, M Antonia Iparraguirre, M Isabel Irizar, M Rosario Larrea, M Dolores Lopez, Petra Pacheco, M Yolanda Porres, Begoña San Juan, M Aranzazu Suquia, M Teresa Arrospide, Carolina Diez, M Arantxa Igartua, Oihana Jauregui, Alazne Saizar, M Jose Tilves, M Lourdes Etxeberria, M Aurora Valdivielso, Xabier Mugica, M Mercedes Lasagar, Coro Zabaleta.

*Management Bilbao District*: Jesus Larrañaga, Maribel Romo, Pilar Isla. *La Merced Health Center*: M Isabel Urcelay, Mary H Corrales; M Angeles Crespo, Javier Jose M Jesus de Ordozgoiti, M Iciar Elguezabal, Susana Esteban, Catalina Frau, Laura Gallo, Ines Yolanda Martin, Nerea Ordorika, Jose Ramon Perez, M Begoña Relloso, M Soledad Sangroniz, M Iluminada Santos, Patxi Xabier Iturbe.

*Management of Interior District*: Enrique Maiz, Cristina Domingo, Carmen Esparta.

*Matiena Health Center*: Esther Gorostiza; M Esther Azpitarte, Bixente Barrutia, Amaia Bengoa, Francisco Jose Miguel, Ana Isabel Etxebarria, M Belen Garcia, M Jose Ibars, M Jose Lasa, M Carmen Martinez, Maura Pernudo, Lourdes Oribe, M Dolores Ustarroz, Valentina Camino, Leire Corpion, Leire Ortuondo, M Carmen Lopez, Rosana Abraldes, Eneko Ibarruri, Javi Alonso.

*Management of Uribe District*: M Luz Marques; Encarnacion San Emeterio, Anton Elorriaga. *Sondika Health Center*: Enrique de la Peña; M Carmen Artola, Teresa Casado, Jesus Garcia, M Paz Sanchez, Luisa Santos, Maria Lanzarote.

Department of Health of the Basque Government: Concha Castells; Francisco Cirarda, Henar Ortuondo, Pilar Manrique, Ines Urieta, Amaia Ajuria.

Clinical Committees:

*Physical Activity*: Ricardo Ortega, Jesus Torcal, M Sol Ariestaleanizbeaskoa, Veronica Arce, Alvaro Sanchez, Gonzalo Grandes; *Diet*: Bittor Rodriguez, Pilar Amiano, Esther Gorostiza, Enrique de la Peña, Alvaro Sanchez, Gonzalo Grandes; *Smoking cessation*: Esther Azpitarte, Mary H Corrales, Josep Cortada, Alvaro Sanchez, Gonzalo Grandes.

Community:

*Beasain:* Juan Manuel Elosegui (Arcelormittal company); Iñaki Korta, Ainhoa Irastorza, Leire Makibar (CAF company); Jon Alkaiaga, Karmele Alkaiaga (Antzizar sports center). *La Merced:* Iñaki Aldamiz (Community Health of Bilbao´s Council); Virginia Zelaia (School Health of Bilbao´s Council); Adela Etxeberria, Itziar Basurto (Miribilla Primary Education Center); Gonzalo Casado, Maite Martinez, Alberto Diez (Bilbao sports center); Javier Rojo (Municipal Office of Bilbao La Vieja, San Francisco and Zabala); Sara Garteiz ("Bakuba" Community association); Aitziber Artabe, Ainhoa Parra ("Etorkinekin Bat" Community association); Marcelo Borja, M Carmen Jimenez ("Iniciativa gitana" Community association*). Sondika:* Gorka Carro, Bernardo Valdivielso (Sondika Council); Janire Kasuso, Esther Martin (Commonwealth municipality of Sondika); Paulino Parra (Gorondagane Primary Education Center); Loinaz Albizu (Txorierri Secondary Education Institute); Marivi Cuartango (Txorierri polytechnic); Unai Atxa (Sondika sports center); Jesus Miguel Enriquez (Olarra company); Rosario Acebal, Javier Ancel (Sondika Pharmacy). *Matiena:* Jose Luis Navarro, Inmaculada Zapardiez (Matiena Council); Nerea Lejarzaburu (Commonwealth municipality of Abadiño); Edurne Madariaga (Traña Matiena Primary Education Center); Eugenia Peral (Abadiño Secondary Education Institute); Pablo Mas (Abadiño sports center); Juan Mayor (Mutualia company); Joseph Reverte (FREMAP company); Bernard Mandaluniz (Estampaciones Metalicas company)

*Others:* Roberto Nuño (Basque Institute for Healthcare Innovation O+berri); Josu Llano (Osarean); Enrique Gutierrez (Osatek SA).
